# Can alternative nicotine products put the final nail in the smoking coffin?

**DOI:** 10.1186/s12954-022-00722-5

**Published:** 2022-12-01

**Authors:** Karl Fagerström

**Affiliations:** Fagerström Consulting, Stockholm, Sweden

**Keywords:** Tobacco control, Harm reduction, E-cigarettes, Oral tobacco-free nicotine products, Snus, Heated tobaccco products

## Abstract

This review describes the evolution of smoking prevalence in countries with relatively high adoption of alternative nicotine products such as e-cigarettes, heated tobacco, and snus compared to neighboring countries where these products are less prevalent. The data indicate that countries with high adoption of alternative nicotine products have been able to achieve lower smoking rates. The findings suggest that adoption of alternative nicotine products may help in reduce smoking prevalence faster than traditional tobacco control measures solely focused on prevention and cessation.

## Background

A recent commentary by Beaglehole and Bonita posited that tobacco control is not working for most of the world [1]. Given that the overall number of tobacco users has barely changed in the last three decades, they recommended that the World Health Organization (WHO) Framework Convention on Tobacco Control (FCTC) incorporate strategies designed to reduce the harms caused by burnt tobacco by replacing cigarettes and other combusted tobacco products with non-combusted alternative products that deliver nicotine in far less harmful ways.

Indeed, as succinctly summarized by Vaughan Rees, “the best, evidence-based interventions are dated, overrated and cannot meet the challenge of reducing tobacco-related harm in this century [2].” Despite a growing awareness that current approaches are falling short, very few countries have adopted harm reduction policies as part of their tobacco control strategy. Notable examples include United Kingdom [3] and New Zealand [4], which explicitly encourage the use of vaping for smoking cessation and harm reduction. Alternative nicotine products have also seen significant uptake in Sweden, Norway, and Japan, which do not necessarily endorse their use to reduce smoking-related harms.

## Methods

This review examined the trends in smoking prevalence in countries with relatively high adoption of alternative nicotine products—the UK, Sweden, Norway, New Zealand, and Japan—and compared them with neighboring countries with lower uptake of these alternatives, namely the average of the 27 countries that comprise the European Union (EU27), Denmark, Finland and Australia. Most of the data were collected from large representative national surveys used for reporting smoking prevalence to the WHO [5].

## Results

**Fig. 1:**
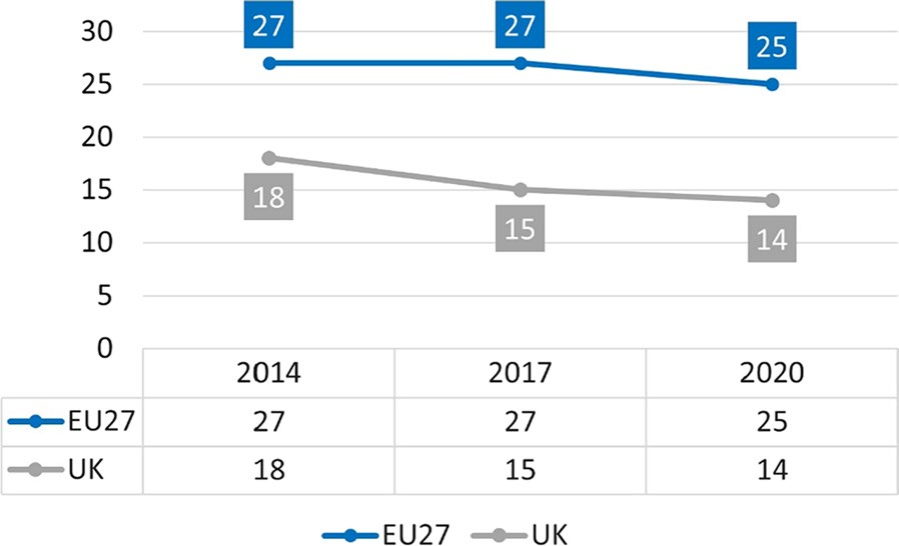
Current smoking prevalence rates in the EU27 [6] (persons aged ≥ 15 currently smoking cigarettes, cigars, cigarillos, or a pipe) and the UK [7] (persons aged ≥ 18 smoking cigarettes)

The 2020 smoking prevalence in the UK was slightly under 14% and appears to have declined faster than in the EU27 in recent years, despite already being far lower in 2014 (Fig. 1). The prevalence in the EU27 dropped by 2 percentage points between 2014 and 2020 compared to a drop of 4 percentage points in the UK. The prevalence of current e-cigarette use in the UK was 6.4% (persons aged ≥ 16) compared to 2% in the EU27 (persons aged ≥ 15).

**Fig. 2:**
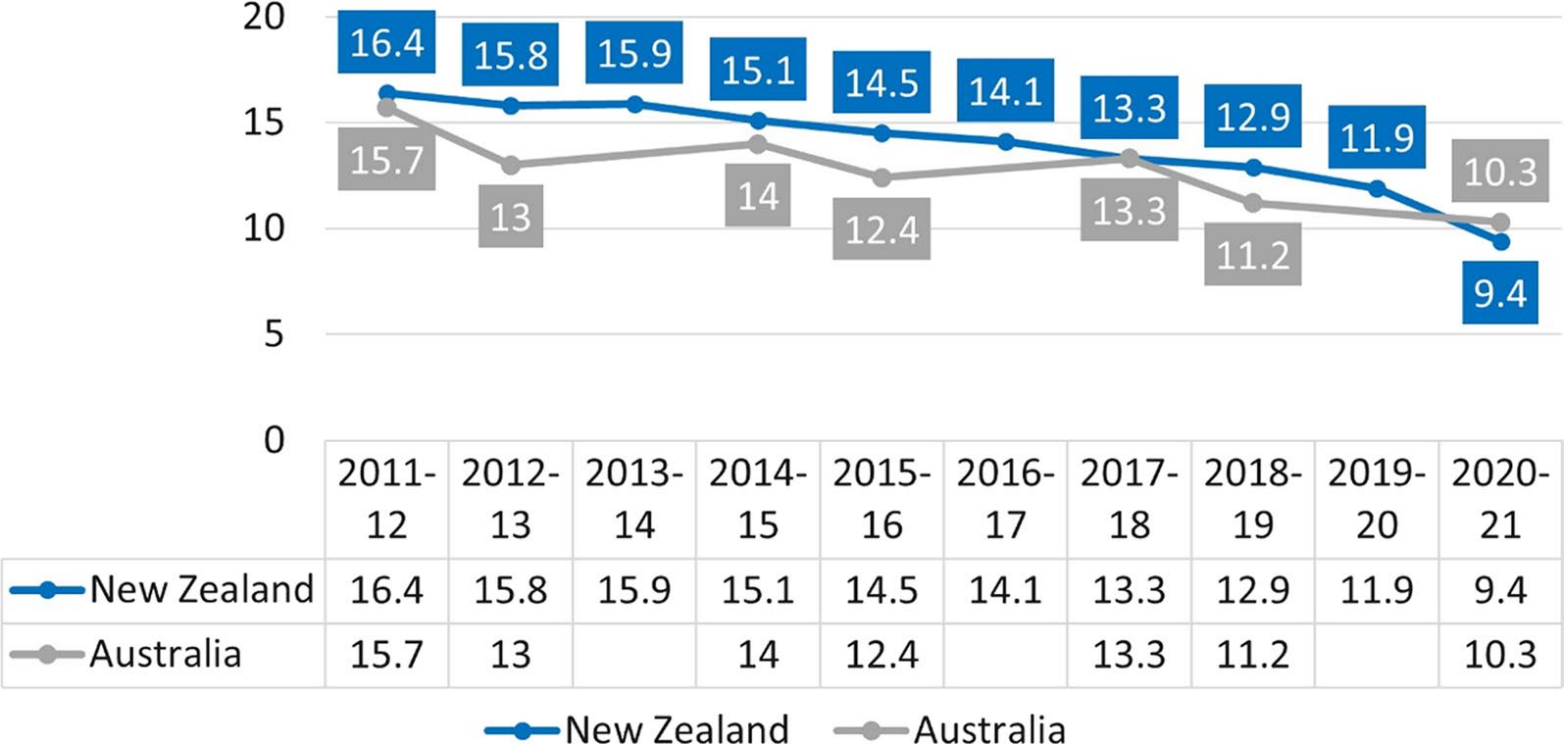
Daily tobacco smoking in New Zealand [8] and Australia [9] (persons aged ≥ 15). *Note*: Data for Australia for years 2011–12, 2014–15, 2017–18, and 2020–21 come from the Australia Health Survey, years 2013, 2016, and 2019 come from the National Drug Strategy Household Survey. The latter survey is used for reporting smoking prevalence to the WHO

In New Zealand, daily smoking prevalence appears to have fallen faster than in neighboring Australia (Fig. 2), even though both countries have similar tobacco control measures for cigarettes (high excise taxes, plain packaging, point of sale display ban). Daily e-cigarette use was estimated at 6.2% (persons aged ≥ 15) in New Zealand in 2021, compared with 1.1% in Australia in 2019 (persons aged ≥ 14). While New Zealand government actively encourages vaping to quit smoking, Australia does not.

Notably, all three countries (the UK, Australia, and New Zealand) prohibit the sales of snus, a low-toxicant oral tobacco product, despite the fact that the availability and use of snus has contributed to Sweden’s record-low prevalence of smoking and the lowest level of tobacco-related mortality among men in Europe [10]. This phenomenon is sometimes referred to as the “Swedish experience.”

**Fig. 3:**
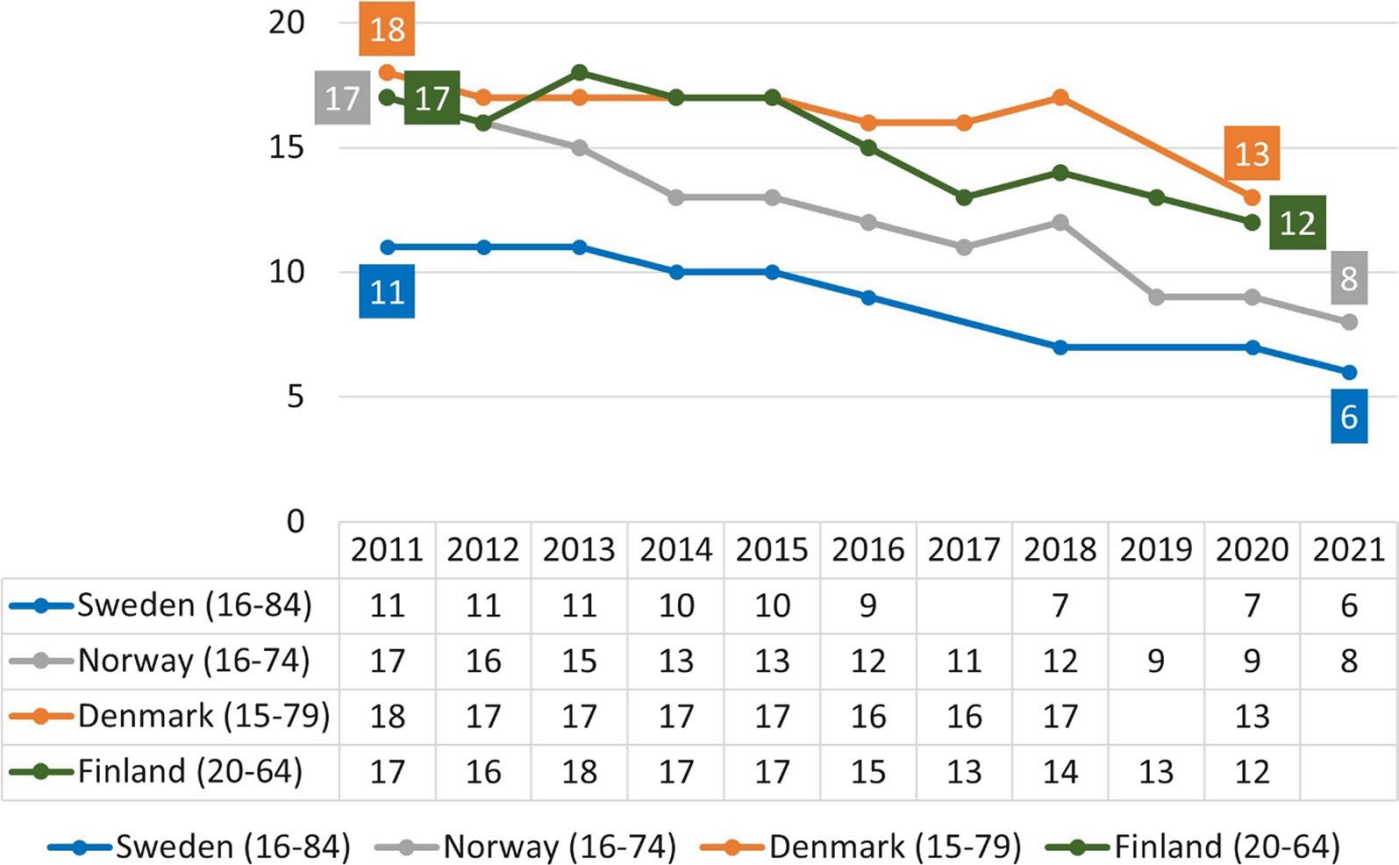
Daily smoking prevalence in Sweden [11], Norway [14], Denmark [15], and Finland [16]

There has been a similar transition from tobacco smoking to snus use in Norway: the daily smoking prevalence has reached almost the same level as in Sweden, while the use of snus is similar. Both countries are far ahead of their neighbors Denmark and Finland in achieving a smoke-free society (Fig. 3). Daily snus use in Sweden was estimated at 13% in 2021 [11] (ULF survey: 15%) [12] versus 15% in Norway [13], far higher than in Denmark and Finland where these products are prohibited. The data shown in Fig. 3 for Sweden are from the National Public Health Survey that has a larger sample size than the Survey of Living Conditions (ULF), which is used for reporting to the WHO. The ULF survey estimated that smoking prevalence in Sweden among persons aged 16–84 was just under 10% in 2021 [12].

**Fig. 4:**
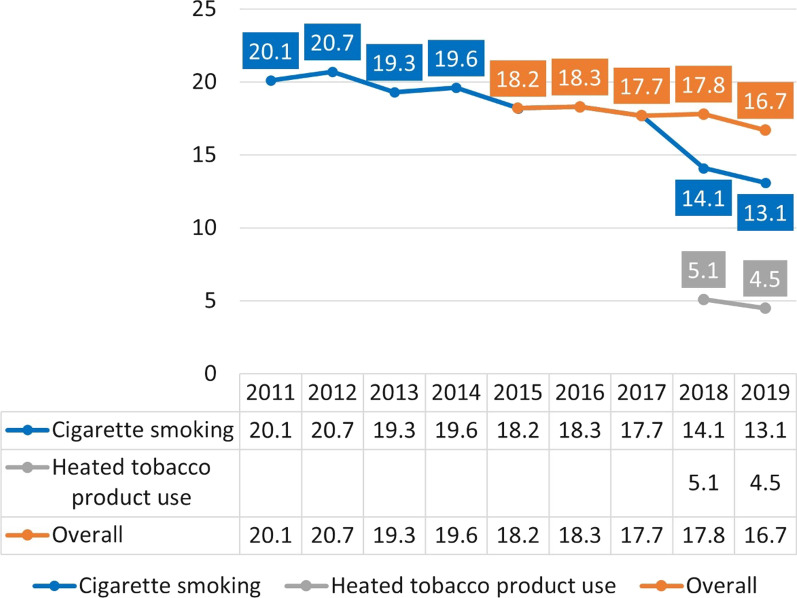
Cigarette smoking and HTP use every day or some days among adults aged ≥ 20 in Japan [17]

It also appears that an even more recent “Japanese experience” is developing with heated tobacco products (HTPs) successfully replacing smoking in this country (Fig. 4). While HTPs have been meaningfully present in Japan since 2015, the Health and Nutrition Survey was only updated to capture the use of HTPs separately from cigarette smoking in 2018. It is therefore likely that HTPs contributed to the lower smoking prevalence prior to 2018. Importantly, the survey also found that ~ 76% of HTP users in Japan were not smoking cigarettes in 2019.

**Fig. 5:**
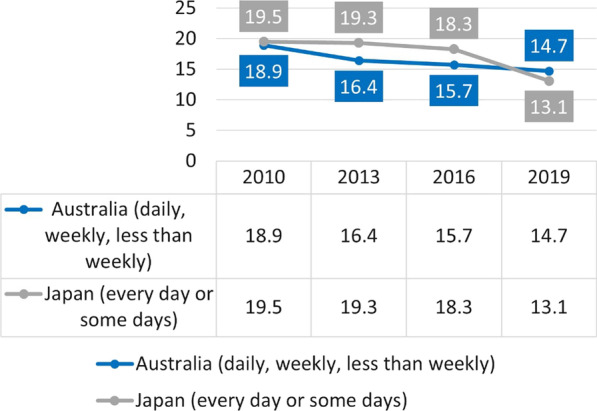
Cigarette smoking prevalence in adults (persons aged ≥ 20, Health and Nutrition Survey) and tobacco smoking prevalence in Australia (persons aged ≥ 18, National Drug Strategy Household Survey)

Interestingly, the current prevalence of cigarette smoking in Japan in the adult population (13.1%) is even lower than in Australia (14.7%), despite far stricter tobacco control policies in the latter country (Fig. 5).

## Discussion

The data reviewed in this paper indicate that substitution of combusted tobacco products with alternative nicotine products may contribute to lower smoking rates. This is supported by the lower smoking prevalence in countries with relatively high uptake of alternatives compared to neighboring countries with lower use of these products. The findings indicate that more rapid adoption of alternative nicotine products may help reduce smoking prevalence faster than traditional tobacco control measures focused on prevention and cessation alone.

While the debate on the degree of the risk reduction of various non-combusted alternative nicotine products continues [18], it is virtually certain that none of these products is anywhere close to smoking in terms of harm. Some of the products from all three categories—snus, HTPs, and e-cigarettes—have also been reviewed by the U.S. Food & Drug Administration (FDA) and found to be “appropriate for the protection of public health [19].” While the FDA has yet to make a determination on premarket tobacco product applications for the most recent product category of nicotine pouches, the product chemistry data indicate that their risk profile is likely similar to nicotine gum, a nicotine replacement therapy that has been on the market for decades [20].

One reason why smoking prevalence has declined in the countries included in this review may be related to the fact that the younger generations are leading the transition away from cigarettes. An exception is Japan, where youth use of all tobacco products is extremely low and tobacco use initiation probably occurs at a later age [21].

Just 1% of Norwegians aged 16–24 smoked daily in 2021 [14]. In Sweden the daily smoking prevalence among 16–29-year-olds was 3% [11], while in New Zealand the daily prevalence of smoking among 14–15-year-old students reached 1.3% [22]. Similarly, past-30-day cigarette smoking among high-school students in the United States was 1.9% [23]. Conversely, the average prevalence of daily smoking among 15–16-year-old students in the 35 countries surveyed as part of the 2019 European School Survey Project on Alcohol and Other Drugs (ESPAD) was far higher at 10%, led by Bulgaria at 21.8% and followed by Croatia (19.5%), Italy (18.7%), Romania (18.4%), and Slovakia (18.3%) [24].

It is understandable that we feel uncomfortable with youth using any nicotine or tobacco, especially as declines in smoking in all these countries have been accompanied by increased use of snus or e-cigarettes. However, as Action on Smoking and Health New Zealand director Deborah Hart explained in the context of achieving low levels of smoking among youth in New Zealand: “this is the biggest fall in youth smoking rates in a decade, and it’s extremely encouraging to see young people leading the progress towards a smokefree Aotearoa [22].”

The reality is that adolescents will probably continue to engage in risky behaviors, regardless of the legal status of a given product or activity. While some 19.6% year 12 students in the United States (aged 17–19) vaped nicotine in the past 30 days in 2021, 19.5% used marijuana, 25.8% used alcohol, and 15.5% reported being drunk at least once in the past month [25]. Therefore, a more pragmatic approach may be to ensure that if teenagers engage in risky behaviors, those risks are minimized.

If current trends persist, it is likely that in countries that have reached very low levels of smoking among youth and young adults, smoking will virtually disappear in one or two generations as these cohorts reach adulthood. However, more could and should be done to reach this objective even faster and in more countries.

The UK and New Zealand prioritize vaping for smoking cessation and harm reduction [3, 4] but are more lukewarm towards other alternatives. On the other hand, while HTPs are permitted in Japan, e-cigarettes with nicotine are not allowed for sale outside the medicinal framework, and are therefore de-facto legally unavailable [26]. Snus is prohibited in all the countries reviewed in this article except Sweden and Norway. Finally, nicotine pouches, the most recent promising lower-risk alternative to cigarettes, often remain in a regulatory vacuum.

A limitation with this paper is that the prevalence rates do not allow to take into account the country based differences that prevents firm comparability between countries, also surveys conducted during the COVID pandemic could introduce some confounding.

## Conclusions

Realistically, no single alternative nicotine product category will be able to reduce smoking rates and the associated disease burden. E-cigarette uptake in the UK and US has been leveling in recent years, and smokers may be more likely to consider and completely switch away from cigarettes if other types of non-combusted alternatives were recommended for use by smokers.

The WHO should be leading the way in proposing bold actions to eradicate smoking, but unfortunately it is not. Instead, it is solely relying on the FCTC treaty mechanism and MPOWER measures that have not accelerated global progress in reducing cigarette consumption [27] or tobacco mortality [28]. Combining strong MPOWER measures for smoked tobacco with support for less harmful alternatives may hasten the goal of eradicating tobacco smoking for good. This means implementing far stricter regulations and taxes on combusted products, scaling-up prevention and cessation programs, and simultaneously giving less hazardous products significant regulatory and fiscal advantages compared to cigarettes. These measures could promote alternative nicotine products as harm-reducing options for smokers who do not quit.

What is clear is that the current status quo is unacceptable. While the global prevalence of smoking has steadily decreased over the past decades, concurrent population growth means that the number of smokers has remained virtually unchanged. A 2019 Global Burden of Disease (GBD) study estimated that there were 0.99 billion smokers in the world in 1990, with the number increasing to 1.14 billion in 2019 [29]. In 2021, the WHO estimated that the number of smokers in 2020 was 0.99 billion [30], exactly the same number as the GBD group estimated for 1990.

Clearly, bolder policies—including endorsement of harm reduction—are needed to put the final nail in the smoking coffin.

## Data Availability

Not applicable.
